# Coronary pressure-derived parameters

**DOI:** 10.1007/s12471-018-1128-y

**Published:** 2018-06-25

**Authors:** J. J. Piek

**Affiliations:** Department of Cardiology, Amsterdam UMC, location AMC, Amsterdam, The Netherlands

Fractional flow reserve (FFR) has emerged as an established intracoronary diagnostic technique for the evaluation of coronary lesion severity during cardiac catheterisation. The results of the FAME I and II trials have led to a class IA indication of FFR if evidence of myocardial ischaemia is not available [1, 2]. Despite this recommendation, the use of coronary physiology in daily clinical practice has shown a modest growth over the last decades. To minimise the microvascular resistance FFR requires the induction of maximal hyperaemia, by intracoronary or intravenous administration. Hypothesis is that intravenous induction of maximal hyperaemia in particular is time-consuming and one of the constraints of using FFR. This development led to the introduction of the instantaneous wave-free ratio (iFR), the diastolic pressure gradient across a coronary lesion during baseline conditions. It was assumed that the simplicity of the technique would lead to a more frequent use of iFR for diagnostic purposes. The usefulness of iFR, as compared with FFR, was recently highlighted in the DEFINE FLAIR and SWEDEHEART studies [3, 4]. The use of either iFR or FFR shows that PCI can be deferred in approximately 50% of patients in coronary lesions of intermediate severity, indicating their usefulness. The clinical outcome of an iFR strategy was similar to an FFR strategy. The recently presented sub-analysis of the DEFINE FLAIR study demonstrated the cost-effectiveness of iFR versus FFR due to a reduced number of coronary interventions and a reduction in procedural times [5].

In the current issue of the Netherlands Heart Journal, Pisters et al. [6] studied 356 consecutive patients with 515 coronary stenosis by direct comparison of iFR and FFR. This analysis showed a good correlation between iFR and FFR (*r* = 0.75) and a high area under the receiver-operating characteristic curve (0.92) for iFR compared with FFR. This study confirms earlier studies that showed that iFR may serve as an alternative for FFR. For practical purposes, iFR has the additional advantage that a pull-back curve can be easily recorded to document the segment in question in the diffuse disease that is frequently encountered during cardiac catheterisation.

How should we interpret the data of Pisters et al.? It is overwhelmingly clear that the use of intracoronary physiological parameters adds to the armamentarium of the interventionalist in view of the limitation of coronary angiography when assessing the functional significance of coronary lesions. FFR was introduced as a simple technique and was validated in patients with single-vessel disease and a normal left ventricular function, while unfortunately it is now applied for numerous coronary entities that it was not validated for. The coronary circulation is a flow-driven system and the concept of FFR assumes a linear correlation during hyperaemic conditions between the pressure gradient across a lesion and coronary flow. In subsequent clinical studies, it became clear that the distal microvascular resistance has a marked influence upon the pressure gradient during hyperaemia across a coronary lesion [7]. A high microvascular resistance results in reduced flow across the same lesion, and consequently a high FFR. Vice versa, a low microvascular resistance results in increased flow across the same lesion and a lower FFR. This ‘discordance’ between FFR and coronary flow reserve is a frequent finding and occurs in approximately 30–40% of coronary lesions [8]. The use of iFR for this purpose is less prone to this phenomenon as hyperaemia is not induced. Initial iFR studies used a grey zone for iFR (0.86–0.93) and it was advocated to additionally assess FFR if the iFR was within this grey zone. Unfortunately, this study protocol does not always provide the correct answer. For example, a borderline iFR may result in a low FFR when coronary flow reserve is normal, a so-called non-flow limiting lesion that does not require treatment [9]. This is in line with the results of the FAME II trial showing that 50% of the patients did not need coronary intervention during 5 years of follow-up despite an abnormal FFR, probably because of the inclusion of non-flow limiting lesions [10]. On the other hand, when the distal microvascular resistance is high, a borderline iFR will result in a normal FFR that could probably benefit from treatment as the coronary flow reserve is already exhausted. One has to be aware of these limitations of pressure-derived parameters for correct patient management and it emphasises the need for documentation of myocardial ischaemia prior to intervention. Unfortunately, many patients are admitted to the cardiac catheterisation laboratory without any documentation of myocardial ischaemia because many cardiologists believe that according to guidelines the FFR can be used as a substitute for myocardial ischaemia [11, 12]. As recently discussed in this journal, clinical decision making should be based upon the correct interpretation of the patient’s symptoms and documentation of myocardial ischaemia by non-invasive techniques in combination with the use of the coronary physiological parameters [13]. In that respect, we should not consider FFR as the gold standard, but rather the patient. This means that the patient should benefit from a coronary intervention regarding their symptoms if the decision model suggests treatment and should be safe if the decision model suggests deferral.

The study of Pisters et al. is welcomed because it advocates the application of intracoronary physiological parameters, which are still markedly underused in daily clinical practice. Unfortunately, coronary physiology is complex and coronary interventions should be performed based on appropriate judgment of symptoms, documentation of myocardial ischaemia and correct interpretation of pressure derived parameters such as iFR and FFR.
